# Inequality and private health insurance in Zimbabwe: history, politics and performance

**DOI:** 10.1186/s12939-023-01868-9

**Published:** 2023-03-29

**Authors:** Alison T. Mhazo, Charles C. Maponga, Elias Mossialos

**Affiliations:** 1 Ministry of Health, Community Health Sciences Unit (CHSU), Private Bag 65, Area 3, Lilongwe, Malawi; 2 Department of Pharmacy and Pharmaceutical Sciences, Faculty of Medicine and Health Sciences, University of Zimbabwe, P.O. Box A178, Avondale, Harare Zimbabwe; 3 The London School of Economics and Political Science, Department of Health Policy, LSE Health, London, UK

**Keywords:** Private health insurance, History, Politics, Performance, Agency problem, Universal health coverage, Zimbabwe

## Abstract

**Introduction:**

Zimbabwe has one of the highest rates of private health insurance (PHI) expenditures as a share of total health expenditures in the world. The perfomamce of PHI, known as Medical Aid Societies in Zimbabwe, requires close monitoring since market failures and weaknesses in public policy and regulation can affect overall health system performance. Despite the considerable influence of politics (stakeholder interests) and history (past events) in shaping PHI design and implementation, these factors are frequently sidelined when analyzing PHI in Zimbabwe. This study considers the roles of history and politics in shaping PHI and determining its impact on health system performance in Zimbabwe.

**Methods:**

We reviewed 50 sources of information using Arksey & O'Malley's (2005) methodological framework. To frame our analysis, we used a conceptual framework that integrates economic theory with political and historical aspects developed by Thomson et al. (2020) to analyze PHI in diverse contexts.

**Results:**

We present a timeline of the history and politics of PHI in Zimbabwe from the 1930s to present. Zimbabwe's current PHI coverage is segmented along socio-economic lines due to a long history of elitist and exclusionary politics in coverage patterns. While PHI was considered to perform relatively well up to the mid-1990s, the economic crisis of the 2000s eroded trust among insurers, providers, and patients. That culminated in agency problems which severely lessened PHI coverage quality with concurrent deterioration in efficiency and equity-related performance dimensions.

**Conclusion:**

The present design and performance of PHI in Zimbabwe is primarily a function of history and politics rather than informed choice. Currently, PHI in Zimbabwe does not meet the evaluative criteria of a well-performing health insurance system. Therefore, reform efforts to expand PHI coverage or improve PHI performance must explicitly consider the relevant historical, political and economic aspects for successful reformation.

## Background

“That this hope (accessing health care) is often shattered has been the experience of the Rhodesian State Lottery Trustees in dealing with cases of distress caused by illness—having had some experience in the administration of medical aid societies and having myself greatly benefited through such organizations, I had little doubt that the answer to the problem was the expansion of medical aid societies”

Phillip Nigel, one of the pioneering figures for the development of PHI in Southern Rhodesia (present-day Zimbabwe) writing in South African Medical Journal (1957).

Private health insurance (PHI) offers financial protection from the catastrophic effects of illness [1]. It is provided through the direct payment of premiums to insurers. In contrast, public insurance is typically funded through taxes and either general or earmarked social security contributions [2]. Regardless of form or design, the primary function of health insurance (PHI included) is to provide “access to care with financial risk protection" [3]. The type of PHI that develops in a country and its role are determined by public financing of the country's primary health care system or the “public system" [4]. PHI’s role can be supplementary, complementary, or substitutive [5].

Four aspects of the public system determine opportunities available to insurers for developing a PHI market, also known as the *core drivers of market development* [4]. These include the share of the population entitled to services (breadth of coverage), the benefits covered by the public system (range of coverage), the benefit–cost proportion met by the public system (height of coverage), and consumer satisfaction (perceptions about the quality of care). From a Universal Health Coverage (UHC) perspective, unlike taxation and social security schemes, which are commonly viewed as promoting equity in healthcare financing, private insurance often conjures up visions of unequal access, large numbers of uninsured people, and elitist health care for the rich [6]. However, if properly regulated, PHI can support UHC efforts by reducing out-of-pocket (OOP) expenditures, supplementing public health expenditures, relieving fiscal pressure on publicly funded systems, and acting as a gateway to developing social health insurance systems [6]. Conversely, PHIs can adversely affect the overall health system performance because of the inherent market failures and weaknesses in public policy and regulation [7].

Market failure in PHI arise from the characteristics of medical care as a subject of normative economics. In insurance markets, the theory of diminishing marginal utility posits that risk-averse and utility-maximizing agents (individuals) are willing to pay a positive amount (health insurance premium) when they are healthy in anticipation of compensation in the event of a future illness [1]. The role of health insurance is to level health care service consumption between periods of good health (low marginal utility) and periods of ill health (high marginal utility) [8]. As a subject of normative economics, the medical care market significantly differs from other markets due to the uncertain timing of disease incidence and need for treatment [9]. Typically, knowledge and information in the medical sector are not evenly distributed across an entire population, but concentrated in a few individuals who can potentially profit from it; a  phenomenon referred to as *information asymmetry* [10, 11]. The medical market also differs from “normal markets” in that, although health is valuable, it cannot be sold on the market like other commodities; therefore, individuals seek health care as derived demand for health.

In many health systems, insurers act as intermediaries between the patient and the provider. This relationship is known as a “medical triad” or *relational network* [12, 13]. This relationship distributes risks and incentives among the players in the medical triad, which can give rise to the agency problem. An agency problem arises when any player in the economic arrangement (in this case, any player in the medical triad) seeks to maximize their incentives, typically by shifting risks to others due to different preferences [13, 14]. In the context of health care and health insurance, these risks and incentives (their design, origin, distribution, and the extent of the clash between them) determine who gets what, when, and how; which  forms the heart of healthcare market politics [15]. In turn, the nature of politics profoundly influences the insurance sector and the entire health system [1, 16].

Economic theory posits that agency problems in voluntary forms of health insurance induce market failures, characterized by failure to achieve optimally efficient resource allocation [1, 17, 18]. Two of the primary forms of market failures in health insurance are *adverse selection* and *moral hazard*. Adverse selection hinges on “hidden information or the fact that individuals who seek health insurance know more about their health status than the insurer” [17]. In contrast, moral hazard hinges on “hidden action”—the idea that insurance coverage can increase healthcare use [19]. Moral hazard includes supplier-induced demand when providers use their discretionary influence to over-service patients with unnecessary care [20]; which is a significant problem in fee-for-service arrangements [21–24].

The performance of PHI in Zimbabwe requires monitoring because of its disproportionately high (30%) expenditures as a share of total health expenditures relative to population coverage (10%) [25]. Even though PHI design and performance reflects the influence of history and politics more than informed choice, these aspects are rarely examined in the Zimbabwean context. For a country with a long history of PHI and a health financing system that has been a subject of a rapidly evolving political and economic changes [26], any assessment of PHI that marginalizes the role of politics (stakeholder interests) and history (past events) will fail to provide comprehensive insight into the successes, failures, and challenges associated with PHI performance in Zimbabwe.

This study comprises three parts. First, we briefly examine the historical aspects of health system development in Zimbabwe and provide an overview of health system financing. Next, we discuss the origins and development of the PHI market in Zimbabwe (history) in light of stakeholder interests (politics). Finally, we assess PHI performance in Zimbabwe over time according to the following four evaluative criteria [27]: 1) Can PHI fill gaps in publicly financed coverage? 2) Does PHI provide financial relief for the government? 3) Does PHI enhance access to health care? 4) Does PHI improve efficiency in health service delivery?

### The history of Zimbabwe’s health system

When Southern Rhodesia (present-day Zimbabwe) became a British colony in 1890, the early colonial occupiers dealt with healthcare as part of the broader “native question” [28]. Up to the early 1920s, the health care system was dominantly rudimentary and enclavist, primarily serving the medical needs of the minority population consisting of White colonialists and their African employees through private practitioners who arrived with the Settlers [29, 30]. However, in the aftermath of a landmark transition to a self-governed colony from imperial Britain in the early 1920s, there was mounting pressure to improve the health services available to Africans and create a buffer between the “diseased” African population and the European community [31]. By the early 1930s, rural health facility expansion was prioritized by the new colonial administration due to trans-colonial learning, the emergence of a colonial regime sympathetic to natives’ affairs, and pressure to prove the colony’s capacity for self-governance [32]. In this regard, the development of medical services in Southern Rhodesia was directed by the central colonial administration. This differed from other parts of Africa where pioneer efforts were driven primarily by religious organizations [33].

In addition to drivers of health sector development, structurally, Southern Rhodesia sought to establish itself as the socio-economic hub of the federal arrangement that integrated Southern Rhodesia with Nyasaland (present-day Malawi) and Northern Rhodesia (present-day Zambia). As a result of advantageous socio-economic development, by the 1950s, Southern Rhodesia had a relatively well-advanced public health system compared to its federal and regional counterparts. Patients from Central and East Africa sought specialized services including neurosurgery, cardiothoracic surgery, radiotherapy, and dialysis [33]. However, despite the seemingly notable progress and the rhetoric about building a national health service, services for Africans remained rudimentary, and health care provision was unequal, segregated, and favored the White minority. As the public transformed, a large European minority fostered a vibrant, parallel private practice sector to serve their own needs [31]. When colonial rule ended in 1980, the majority government of independent Zimbabwe inherited a racially divided health care system. The White minority enjoyed sophisticated health care while most Africans relied on sparse and poorly resourced public facilities [34]. The government adopted a socialist political orientation to redress this pervasive colonial legacy by aggressively redistributing health resources to the majority Black population as part of efforts to “democratize” the health sector [35, 36]. However, vested private interests (which favored the White minority) were mostly preserved as part of the racial reconciliation policy [35]. This pattern was further entrenched by a shift to neo-liberal and market economy ideas in the 1990s [34].

### Overview of health financing in Zimbabwe

The health system in Zimbabwe is financed through a mix of public and private sources. Public funds are sourced by the Ministry of Finance and the Municipalities. Private funding sources include corporations, households, non-profit organizations, and donors. The financing sources’ contributions are shown in Table 1, below:

**Table 1:** Financing sources as a percentage of Total Health Expenditure (THE)—2018

Source	% of THE
Government	44.1%
Corporations	15.8%
Households	13.43%
Rest of the world	26.7%

Source: Zimbabwe National Health Accounts 2017 and 2018

Central General Revenue, obtained by the central government from taxes and non-taxable revenue, remains the primary funding source for government THE through fiscal allocation [37]. However, most of this money is directed toward salaries and other employee-related costs (30%), with low capital investment (1% in 2017 and 4% in 2018). As a result, service availability and quality is severely hampered at public health facilities [38] and user fee policy is strictly enforced for consultation, in-patient and out-patient services to cover the deficits.

As shown in Table 1 above, non-government or private sources (corporations and households) are essential health service funders in Zimbabwe, contributing to nearly 30% of Zimbabwe’s THE. The dominance of private funding sources, mainly through household user fees, contributes to high OOP spending as a share of THE. Although OOP as a share of THE declined from 37% in 2010–2011 [39] to 13% in 2018, that decline should be interpreted with caution as it coincided with high rates of forgone care (28%) amidst deteriorating socio-economic conditions and escalating medical inflation [38]. Because healthcare is largely financed by individual households and OOP spending, the incidence of catastrophic health expenditure (CHE) remains high and regressive—7.6% in the general population and 13.36% among the extremely poor in 2015 [25]. Over-reliance on OOP and the regressive nature of health service financing in Zimbabwe is mainly driven by the absence of a publicly funded health insurance system [40]. Despite persistent calls to establish a publicly funded National Health Insurance (NHI) since the mid-1980s [41], to date there has been no meaningful progress on the ground and currently there are no active policy discussions on the issue. Therefore, PHI remains the only prepayment mechanism for accessing health care in Zimbabwe.

According to the World Bank, in the 1980s, Zimbabwe had a relatively advanced and well-performing PHI [known as *medical aid societies* (MAS)] modeled after social health insurance systems common to Latin America and Asia [42]. In this study, and consistent with their classification in Zimbabwe, MAS are considered PHI because unlike in public insurance programs, where funds are channeled through the state or quasi-public social insurance organizations managing general or social insurance taxes, MAS directly deal with employers and employees or individuals, avoiding broker costs but also limiting employee discretion [43]. MAS in Zimbabwe are regulated under the Medical Aid Society Registration Act of 2000 [44]. Still, regulatory enforcement is weak, discretionary [45], and vulnerable to governmental conflicts of interest as both a regulator and provider of PHI [40].

Globally, Zimbabwe has one of the highest rates of PHI expenditure as a share of total health expenditures [6, 7]. More than 80% of PHI expenditures are directed to private sector doctors, pharmacies, hospitals, and providers of ancillary medical services [40, 43]. Most MAS are members of the Association of Healthcare Funders of Zimbabwe (AHFoZ), formerly known as the National Association of Medical Aid Societies (NAMAS). NAMAS was formed in 1969 to address issues like standardizing fees, communicating with medical providers, and registering or accrediting healthcare providers and institutions. AHFoZ is a member-based, not-for-profit organization governed by a constitution and a comprehensive code of ethics. Significant stakeholders in AHFoZ include the Ministry of Health, Private Hospitals’ Association, Zimbabwe Medical Association, Government and Mission Hospitals, and all healthcare providers.

There are five different forms of ownership in the medical aid industry: government, corporate general insurance companies (large companies with controlling shareholders), private, not-for-profit health insurance organizations, urban councils, and provider-initiated ownership [43]. At the time of writing (August 2022), 40 MAS were registered with AHFoZ. The three largest PHI organizations are PSMAS (owned by the government), CIMAS (private MAS), and First Mutual (owned by a group holding company). These societies provide coverage for 90% of all people with medical insurance in Zimbabwe [39]. Zimbabwe’s PHI system has a largely supplementary market role, driven by the perceptions of poor quality and timeliness of publicly financed services [4]. Therefore, in Zimbabwe, PHI coverage is mainly geared towards facilitating faster access to services, greater choice of health care providers, and enhanced amenities [46].

## Methods

This study builds on a published scoping review that used the Arksey and O’Malley [47] framework to analyze the political economy of health financing reforms in independent Zimbabwe (1980–2022) [26]. From the 72 articles included in the previous study, we isolated those that specifically addressed PHI. Thirty articles were included at this step. To capture the events of the colonial period, we conducted a free text Google search using the term “private health in Southern Rhodesia.” We used a Google search since the relevant papers and policy documents from the colonial period were not included in available academic databases. Twenty sources of information were identified from the Google search.

## Findings

### History and politics of PHI in Zimbabwe

The history of PHI in Zimbabwe is characterized by pervasive inequalities driven by privileged politics, race, socio-economic class, and urban bias. PHI was introduced in Zimbabwe in the 1930s by the then colonial administration. The PHI scheme, known as the Public Service Medical Aid Society (PSMAS) was run by the same colonial set up to cater for White government employees. The private sector entered the health insurance market in the mid-1940s to cater to corporate and mainly urban-based employees  and to deal with “cases of distress caused by illness” [48]. As these societies grew in strength and provided additional benefits, efforts emerged to extend membership to the rural population, including farmers and miners. These efforts were actively championed by Ian Smith, who later became Prime Minister of Southern Rhodesia. Despite the public and political appeal of the concept, adequate coverage grew incrementally as MAS warned against the “dangers” associated with rapid market expansion to provide coverage to previously uninsured groups.

Although the government favored PHI expansion to alleviate the problem of rising health costs, there was an immediate caveat that such growth should not impose an additional tax burden on the economy. Despite the bureaucratic and fiscal politics, PHI coverage continued to grow throughout the colonial period but mostly covered the White minority population. After independence, PHI was extended to the formally employed Black population following introduction of medical aid as an employee benefit. From a stakeholder politics perspective, offering a package of health care services previously denied to urban and affluent Blacks (i.e., those within the “economically viable sector”) also sought to diffuse political support and momentum for a publily funded insurance  option [49]. While PHI coverage was dichotomized along racial privilege during the colonial period, structural inequalities persist in independent Zimbabwe, albeit with segregation along various forms of socio-economic privilege. A detailed timeline of the history of PHI development is presented in Table 2, below.

**Table 2:** A timeline of the origins and development of PHI in Zimbabwe: 1930–2022

Colonial period: 1930–1979
• **1930**: Public Service Association appoints a special committee to start the Public Service Medical Aid Society (PSMAS)
• **1939**: Resolution submitted to the Rotary Club of Salisbury advocates for MAS to deal with “cases of distress caused by illness due to unaffordability of health care.”
• **1940**–**1944:** Slow progress to expand MAS due to World War II. Black and Asian teachers are barred from joining PSMAS
• **1945**: The Commercial and Industrial Medical Aid Society (CIMAS) begins operations
• **1946**: The Representative Council of Medical Aid Societies of Southern Rhodesia is formed with a mandate to establish collaborative ties with the Rhodesian branches of the British Medical Association
• **1947**: Matabeleland representatives secede from the MAS
• **1952**: Black and Asian government employees are admitted into PSMAS
• **1953**: Under the leadership of Ian Smith, the Parliamentary Select Committee considers expanding MAS to rural areas
• **The late 1950s–1970s**: Growth is observed in the population covered by MAS
• **The early 1970s**: Concerns are raised over escalating costs for MAS and doctors’ demands for increased reimbursement fees
• **1978:** The short-lived Zimbabwe-Rhodesia settlement government led by Bishop Abel Muzorewa declares that the expansion of medical aid to Blacks is a priority
**Post-independent Zimbabwe: 1980-present (2022)**
• **1980:** Zimbabwean government inherits a dual and racially divided health care system that favors the White minority population
• **In the early 1980s**: Most whites continue to seek membership in MAS. A growing number of dominantly urban and elite Blacks became members of MAS mainly due to mandatory PHI for those with formal employment
• **The late 1980s**: About two-thirds of the resident Europeans, but less than one percent of the African population, possess health insurance coverage
• **The early 1990s**: Weak economic performance and perceived unsustainability of public sector subsidies leads to market-oriented health sector reformation, including participation of private insurers
• **The mid-late 1990s:** There is continued interest in diversifying revenue sources for the health sector in Zimbabwe. PHI is considered a viable alternative
• **The 2000s:** Weak economic performance, unsustainable medical inflation, and escalating reimbursement costs threaten the viability of MAS, which then turn to various market survival strategies. The period also features increased capital flow toward MAS
• **2000:** The Medical Aid Societies Registration Act is created to regulate the societies’ registration and conduct
• **2003**: PSMAS launches Premier Service Medical Investments (PSMI) and begins to implement the “Blue Ocean Strategy” to acquire health facilities through vertical integration. During the same period, CIMAS introduces “managed care ideals.”
• **2003:** The Zimbabwe Medical Association (ZIMA) forms the New Independent National Tariff and Liaison Committee to establish fees, independent of MAS, due to the differences between AHFoZ and ZIMA on fee levels and delays in reimbursements
• **2010–2013:** Notable stabilization in economic performance and growth in the number of MAS. Insurance coverage reached an all-time high of 14% during the period
• **2014–Present (2022):** A deteriorating economic environment and a decline in medical society coverage (to around 10%). The period was marked by escalating and recurrent fee wars between ZIMA and insurers. Further vertical integration between MAS and providers

Source: Authors’ collections of various literature

The following sections examine PHI performance against the evaluative criteria mentioned earlier: coverage, fiscal relief, access to care, and efficiency.

### PHI coverage in Zimbabwe

A critical motivation for establishing and expanding PHI is that it improves gaps in publicly financed health coverage [6, 7]. In Zimbabwe, this argument shaped the formation of PHI systems in the 1930s [48], fostered their early development, and persists into present-day Zimbabwe [50]. During the early years of PHI development, only the European minority were allowed to participate; African and Asian government employees were not allowed to join PSMAS (a scheme for the civil service) for over 20 years (from the 1930s to the early 1950s) [48]. In addition to their racialized exclusivity, PHI coverage also favored urban-based corporate employees. In the early 1950s, rural residents—including miners and farmers—were largely excluded from MAS [48]. In 1953, an all-party select parliamentary committee considered expanding MAS to rural areas. Their report on the topic highlighted the following [48]:

*“Our next problem was considering how we could initiate such a scheme. The most apparent method to us seemed to be to encourage one of the present schemes to expand and cover the country districts. I think the House will appreciate the difficulties attached to a suggestion like this. We had considerable help, particularly from one of the biggest schemes in the country, the Commercial and Industrial Medical Aid Society of Southern Rhodesia. They gave us evidence and were willing to help us in every possible way. Still, they did point out the disadvantages and dangers attached to their throwing their doors open to an entirely new block of people who were not of the kind they already catered for.”*

**Fig. 1:**
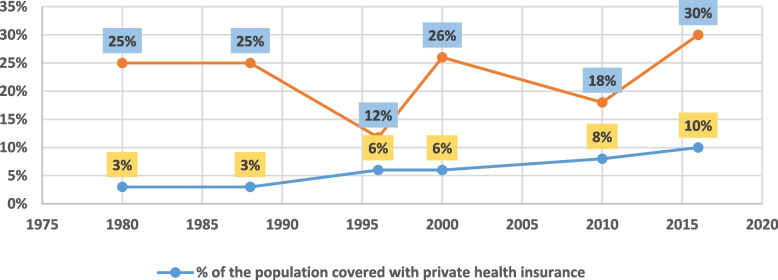
Trend in PHI coverage against expenditures for Zimbabwe: 1980–2016. *Sources: Segall (1983), Manga (1988), Normand *et al*. (1996), Shamu *et al*. (2010), Zimbabwe NHAs (2010, 2015), Zimbabwe National Health Financing Strategy (2017)*

The various dimensions of coverage exclusivity during the colonial period—race, employment status, and geographical dwelling—resulted in negligible coverage that favored privileged individuals. The effect of these coverage dynamics can be best understood in the context of health expenditures. When Zimbabwe gained its independence in 1980, 3% of the population was covered by MAS, accounting for 25% of total health expenditures [51]. In the late 1980s, most Whites (about two-thirds) and a growing number of mainly urban Blacks (~ 1% of the total Black population) were covered by MAS, mainly through corporate plans where employers matched employees’ contributions [41]. MAS coverage still favors the employed and urban-based segments of the population. Figure 1, below, shows the trend in PHI coverage against expenditures for Zimbabwe from 1980–2016.

As shown in Fig. 1, PHI coverage in Zimbabwe grew steadily from 1980 onwards, mainly because of increased participation of Blacks. Although PHI coverage remains very low, it accounts for a disproportionate share of total health expenditures. According to the draft National Health Accounts (NHA) Report of 2017, PHI covered 10% of the population but accounted for 30% of total health expenditures. Thus, a significant percentage of health benefits are concentrated within a minority population segment (mainly urban-based and formally employed). The trend shows fluctuating expenditures relative to a constant population coverage. One of the reasons for the sudden fall in spending in the mid-1990s was the expansion of MAS to government employees in rural areas without the balanced growth of health facilities that accepted medical aid [52].

On the other hand, sudden increases in expenditures could also be related to medical inflation. MAS have attributed expenditure spikes to inefficiencies, including supplier-induced demand, abuse, and fraud [44]. Regarding coverage depth, due to the voluntary nature of PHI participation, population coverage has also been suppressed by the public’s negative perceptions of coverage quality. In particular, the imposition of user fees on individuals covered by MAS as a result of the fee negotiation impasse between MAS and doctors forced some insurered individuals  to drop their coverage in favor of OOP models under the pretext that MAS were of “no use” [53].

Despite the coverage challenges, over the years, MAS have retained features that promote risk-sharing and cross-subsidization in coverage. First, contributions are community-rated (premiums are not determined according to individual risk). Second, the design protects heavy-care users from premium inflation since MAS do not use experience ratings (determining premiums based on previous claims). However, the merits of these equity-enhancing features are countervailed by the existence of multiple insurance pools and various benefit packages offered by the same insurance provider (separated by the ability to pay). As a result, cross-subsidization is effectively constrained due to fragmented pools across different insurers and within–insurers. Additionally, some MAS prohibit high risk groups such as individuals over 65 years old to join their schemes. Nevertheless, MAS have provided financial protection to some of their members, particularly those who can afford plans with generous, high-value benefits.

### PHI and fiscal relief in Zimbabwe

There is no clear evidence that PHI provides fiscal relief to the government of Zimbabwe. Historically, the private sector received hidden subsidies from the government, which sifted resources from the public to the private sector [50, 52, 54]. First, MAS are treated as non-profit entities and are therefore exempted from paying corporate tax under the pretext that the revenue they generate will be re-invested for service improvement. Second, employer contributions to MAS are subsidized through employee premium tax deductions as an incentive to participate in voluntary systems. Third, MAS historically reimbursed government facilities at rates below actual service costs due to weaknesses in the public billing system [52]. In the mid-1990s, this suite of private sector subsidies consumed 12–16% of the government’s health budget but benefited < 10% of the population [50]. Therefore, on top of the disproportionately high share of PHI spending compared to population coverage, various forms of government subsidies further concentrate the benefits on the privileged minority population.

### PHI and access to care in Zimbabwe

Before the economic meltdown of the 2000s, PHI enhanced access to care for those insured and offered a high degree of financial protection. In the mid–1990s, co-payments were rare; which offered an additional degree of financial protection [50]. However, this enhanced access to care and financial security was not equitable. For example, most PSMAS members who are rural government employees could not use their medical aid because there were no facilities that accepted medical aid  nearby [52]. Consequently, access to care and financial protection was biased toward the wealthier, urban-based population.

Given that the other major insurer (CIMAS) membership is mainly corporate based, rural marginalization cannot be excluded. However, since the early 2000s, health facility acquisition by PSMAS through its investment arm PSMI has expanded geographical access to health care among previously marginalized rural civil service members. At the time of writing (August 2022), PSMI owned 126 service centers strategically spread across the 10 geographical provinces of Zimbabwe.

We must also examine coverage quality (the degree to which health insurance enhances financial protection). PHI coverage quality has declined since the 2000s. While co-payments rarely existed until the mid-1990s, they emerged in the 2000s amid a deteriorating economic environment and a financial crisis. First, the traditional collaborative relationship between insurers and providers could not withstand the pressures of an adverse operating environment. From its inception, early medical aid industry creators in Zimbabwe realized the importance of establishing harmonious ties with providers. The Representative Council of Medical Aid Societies of Southern Rhodesia was formed in 1946 with a mandate to establish fees with the Rhodesian branches of the British Medical Association [48]. Until the mid-1990s, MAS agreed on fees with private care providers. Most payments for hospital services followed a government-approved price list known as the Relative Value Scale (RVS) [50]. In 2003, the long tradition of fee agreement between providers and insurers deteriorated when the Zimbabwe Medical Association (ZIMA) formed the New Independent National Tariff and Liaison Committee to create and set prices independent of MAS due to the differences between AHFoZ and ZIMA on fee levels and reimbursement delays. This dual fee level meant that, in reality, insurers set reimbursement fees lower than the liaison committee. In turn, providers made up for the fee changes by levying co-payments on consumers. Second (and worse), some providers ultimately rejected certain MAS, passing all medical costs onto patients [40]. Thus, those with insurance coverage were still burdened with OOP payments either as a cost-sharing model or were treated as if they were uninsured. The fee impasse and its downstream implications is the main reason why some citizens perceive that medical aid is of no use, and instead save money for medical necessities [53]. Among the insured, there is a gap between expectations and actual service delivery. This induces general dissatisfaction across all customer experience dimensions: reliability, responsiveness, service assurance, empathy, and tangibles [55]. Third, the harsh economic environment forced MAS to engage in various cost-containment measures, including reimbursement caps. The introduction of reimbursement caps caused some members to exhaust their annual coverage limit, forcing them to revert to OOP payments. This outcome disproportionately disadvantaged heavy-care users and members with basic (low) coverage levels.

### PHI efficiency in Zimbabwe

In the 1980s, Zimbabwe's PHI was considered relatively efficient compared to similar systems. While efficiency (or lack thereof) is intrinsically hard to measure and quantify within the health sector, integrating empirical evidence and economic theory provides a platform for pinpointing and predicting efficiency-related aspects. Historically, the relatively stable economic environment enhanced efficiency by allowing MAS to exercise leverage over health care providers through agreed-upon fees and implementation of the Relative Value Scale. Up to the mid-1990s, MAS providers effectively faced a single buyer who could use their monopsony power to exert downward pressure on the costs of services [50]. However, as noted earlier, the monopsony power was eroded into the 2000s due to fragmentation in the fees paid to service providers.

Regarding cost containment, MAS historically benefited from inherent weaknesses in the insurance coverage amongst the rural population and inefficiencies in the public system [52]. Insured rural civil servants' limited use of medical aid cushioned MAS from reimbursing legitimate consumption. On the other hand, public sector billing system inefficiencies and limited debt collection capacity allowed MAS to retain all unclaimed funds. It is also worth noting that from inception, providers in Zimbabwe are paid on a fee-for-service basis. This makes them vulnerable to various forms of inefficiency and wastage, including supplier-induced demand. While there is no empirical evidence to quantify the existence of such practices, MAS justified insurer-provider integration to address problems related to overcharging, self-referral (doctors claiming more money on reviews), over-servicing, fraud, and abuse [44]—reinforcing sentiments that surfaced in the early 1970s [56]. The other efficiency-related dimension of interest concerns the share of administrative costs as a percentage of PHI expenditures. In the mid-1990s, administrative costs for PHIs ranged from 8–12% and there was little evidence of systematic “cream skimming” of risks [50]. However, since the 2000s, there has been a rapid increase in administrative costs, suggestive of inefficiencies. According to the Zimbabwe National Health Accounts Report of 2010, private MAS spent 56% of their financial resources on administration, and the remaining 44% accounted for subscriber benefits. In contrast, the National AIDS Council (through the AIDS Levy), spent 17% on administration and the rest on service provision [39]. For some MAS, high administrative costs have been attributed to excessively lucrative salaries and perks for top executives amid poor service delivery [57]. According to the Zimbabwe National Health Accounts of 2015, administrative costs for PHIs had declined to ~ 17% [25], much lower than previous periods but considerably higher than the conventional belief that administration costs should not exceed 10% of total spending [58].

## Discussion

This scoping review found that the development and performance of PHI in Zimbabwe had been heavily influenced—and continue to be influenced—by the interaction of politics (stakeholders’ interest) and evolving political economy conditions (history). Table 3 below summarizes PHI performance in Zimbabwe according to the pre-defined evaluative criteria.

**Table 3:** Evaluation of PHI performance in Zimbabwe

Evaluative criteria	Performance
Can PHI fill gaps in publicly financed coverage?	• Deficient population coverage • Coverage in favor of urban-based and formally employed individuals • Coverage according to wealth index • Individualized expenditures similar to medical savings accounts as citizens opt out of PHI
Does it enhance access to health care or improve efficiency in health service delivery?	• Financial barriers to care as insured individuals incur OOP expenditures • Geographical barriers to care for rural-based enrollees • Financial protection for insured individuals with generous coverage benefits • The dominance of fee-for-service payment mechanisms predisposes the system to inefficiencies driven by a moral hazard such as supplier-induced demand • High administrative costs as a share of total expenditures • Disproportionately high expenditures relative to the proportion of the population with coverage
Does it provide financial relief for the government?	• The private sector sifts resources from the public sector through direct and indirect government subsidies

### Population coverage

Population coverage remains dismally low because PHI enrollment is structurally designed to favor privileged individuals. While enrollment on PHI in the colonial era was based on institutionalized racism, wealth, and employment status became the basis of enrollment in independent Zimbabwe. These historical shifts mirror South African patterns during and post-apartheid [59]. Currently, PHI coverage in Zimbabwe is influenced by the wealth index, as with South Africa, Nigeria, Togo, Benin and Mali, and Madagascar [60, 61]. Since Zimbabwe does not have publicly funded health insurance, it is predominantly the elite who can access health care using prepayment mechanisms. Unsurprisingly, OOP expenditure in Zimbabwe remains a dominant financing mechanism, with catastrophic consequences concentrated on the poor [25, 62, 63]. By relying on PHI as the dominant prepayment mechanism for health, a large segment of the population would remain uncovered, undermining the core UHC tenets of solidarity and cross-subsidization [64].

Furthermore, willingness to enroll in PHI was severely affected by the breakdown of trust or social capital [65] in the “medical triad” (providers, insurers, and patients). Particularly harmful is the perception that OOP payments are necessary, even for insured individuals. The outcome mimics medical savings accounts as individuals opt out of PHI in favor of individualized expenditures. This model severely undermines risk pooling and population cross-subsidization [66, 67]. As Zimbabwe seeks to expand PHI or establish other forms of publicly funded insurance systems, the focus should not be solely on the technical aspects of reform; instead, restoration of trust should be considered a fundamental precursor to such reforms to enhance implementation feasibility. Social capital is key to the success of health insurance systems in developing countries [68], including Zimbabwe, where willingness to pay for NHI has been threatened over concerns about transparency and accountability [43]. Once a health insurance model is in place, the role of trust in combating agency-related problems must be seriously considered  [11, 69].

### Fiscal relief

PHI does not contribute to fiscal relief in Zimbabwe. Historically, the government of Zimbabwe has extended a range of incentives and subsidies to stimulate PHI uptake, similar to countries such as Australia and Ireland [70–72]. Considering that PHI covers a minority and relatively well-off segment of the population, these subsidies were inefficient and inequitable as they drew financial and other resources away from the public sector and the people who needed them most [43]; which mirrors international concerns [70, 71]. In contrast, the publicly oriented National AIDS Trust Fund (AIDS levy) enabled strategic fiscal relief by filling publicly funded gaps to achieve a multi-sectoral response to HIV/AIDS, particularly for the procurement of anti-retroviral (ARV) medicines [73]. To the extent that the Zimbabwean experience directly contradicts the common notion that PHI fills gaps in publicly funded systems, it also demonstrates that PHI design heavily influences the public sector, and vice versa. As a result, and from a public policy perspective, any reform that seeks to introduce a publicly funded insurance system should consider existing and anticipated interactions with the private sector. Of particular importance is how incentive-driven private sector interests can dampen the prospects of publicly funded insurance systems, as happened in Zimbabwe in the 1980s [41], and in the 2000s for South Africa [74] and Uganda [75]. Therefore, proponents of health insurance reforms in Zimbabwe should not overly rely on technical merits to advocate for reforms, but rather consider the influence of stakeholder interests (politics). Though underutilized, this approach can help explain health insurance reforms in developing countries [76]. Another public policy issue of importance concerning the nexus between health financing reforms and the fiscal environment is how the political economy of donor funding potentially interacts with broad-based UHC reforms such as health insurance. Considering that health aid is fungible and not entirely additional [77], large in-flows of development assistance toward tuberculosis, HIV/AIDS, and malaria allocated to Zimbabwe since the early 2000s could have displaced domestic resources and dampened the urgency for health insurance reforms amidst concerns of donor dependency [78].

### Financial protection and access to health care

PHI does not effectively and consistently offer financial protection since enrolled individuals incur various forms of OOP expenses. We posit that the erosion in economic security, beginning in the 2000s, is rooted in the worsening of the agency problem that is in line with corporate behavior under economic wide-financial crisis [79]. Facing a hyperinflationary environment and delayed payments by insurers, providers behaved as rational economic agents by shifting the risk to the insurer and charging higher rates to cushion themselves from income erosion (preservation of incentives) or levying user fees. Reimbursement delays require careful attention since they act as disincentives for service providers to effectively collaborate with insurers in other developing countries [80, 81]. Since enrolled individuals incurred OOP payments, the intertwining agency problems in Zimbabwe threatened the foundational principles of health insurance: the inter-temporal smoothing of health care consumption. As Zimbabwe seeks to expand prepayment health expenditure mechanisms, the focus should consider *both* quantitative components that emphasize population coverage *and* coverage quality. In other words, expanding insurance schemes and achieving population coverage is useless if the population does not access the needed services or incur OOP payments at the point of consumption. Therefore, development of a public insurance system in Zimbabwe would not guarantee access to health care since the demand for health insurance is derived from the health care market. Therefore, to motivate insurance system development, a parallel effort must be directed towards building the health system and improving the performance of existing prepayment arrangements. We also wish to highlight constraints to adequate PHI coverage in Zimbabwe. These occur as a result of hostile economic conditions, agency problems, and a changing disease landscape. At the peak of the HIV/AIDS epidemic in the 1990s, PHIs could not fully cover the high treatment costs due to sustainability concerns [82]. This highlights the dilemma of balancing the breadth, scope, and depth of health insurance in the context of UHC; who is covered, what services are covered, and to what extent service-related costs are covered [83] Therefore, as Zimbabwe seeks to expand prepayment financing mechanisms, due consideration should be directed towards  technical designs that would ensure the subsidization of high-cost patients to balance financial protection and sustainability, particularly in the context of rising non-communicable diseases and cost-intensive conditions such as cancer.

### Efficiency

The efficiency-related aspect of PHI performance has also deteriorated due to economic problems. In the early 2000s, insurers claimed 20– 30% of health resources were wasted due to the pervasive moral hazard form of supplier-induced demand, fraud, and abuse [44]. Concerns about moral hazards in health insurance have also been raised in Ghana [84–86]. As stated earlier, a fee-for-service payment mechanism (the traditional payment mechanism in Zimbabwe) is most vulnerable to inefficiencies such as supplier-induced demand [20, 21]. While MAS intervened by introducing the ideals of managed care, they did not explore other managed care concepts, such as the transition from fee-for-service to capitation, to protect their relationships with providers and beneficiaries' employers [44]. Despite the expressed urgency for provider payment reforms, capitation has not been discussed as a serious policy consideration in Zimbabwe and has never been implemented. This reflects the influence of path dependency in Zimbabwe’s PHI reform efforts [87–89]. In principle, prospective payment methods such as capitation are designed to address the agency problem by shifting some of the risks to the provider and are therefore vulnerable to stakeholder resistance to the extent that such reforms can “fall off the policy agenda,” as in the case of Ghana [84, 90]. Considering the long fee-for-service tradition in Zimbabwe, and the fact that PHI contributes 80% of private sector income, the experiences of countries like Ghana provide key lessons on the technical and political obstacles associated with “departing from the path” if such provider payment reforms are considered. As Zimbabwe seeks to expand insurance systems, careful consideration should be directed toward the initial technical design of such arrangements, particularly since future policy changes will likely be met with resistance due to “incentive lock-in” among stakeholders, even if the initial design was sub-optimal.

## Strengths and limitations of this study

This scoping review was subject to some limitations. First, we may have missed some relevant studies due to limited database selection, time constraints, and the non-inclusion of sources published in languages other than English. Second, we did not appraise literature quality. Despite these limitations, this study is the first to examine the origins and performance of PHI in Zimbabwe within a historical lens that dates back to the pre-colonial period. This is also the first analysis of PHI in Zimbabwe to apply economic theory with a focus on market failures and agency problems. By integrating structural dimensions and economic theory, we hope to have built upon traditional descriptive analyses of PHI performance in Zimbabwe to unpack the underlying performance drivers.

## Conclusions

In Zimbabwe, PHI fails to achieve any of the four criteria we applied to its performance evaluation: adequate coverage, providing fiscal relief, enhancing access to care, and promoting efficiency in service delivery. The history of exclusivitythe politics of divergent stakeholder interests, and agency problems in light of economic issues continue to influence current coverage performance, and it is likely to be so unless relevant reforms are implemented. Future PHI and health insurance reform efforts should prospectively manage stakeholders' interests to facilitate implementation of desired reforms. On the other hand, proponents of such reforms should consider the influence of history and the risks inherent in creating new actors and institutions that may be difficult to direct and impossible to dismantle. Direct PHI reforms, or the establishment of other UHC-oriented institutions like an NHI system, require restoring social capital among stakeholders in Zimbabwe. 

## Data Availability

The current study's datasets are available from the corresponding author upon reasonable request.

## Abbreviations

AHFoZ: Association of Healthcare Funders of Zimbabwe
CHE: Catastrophic health expenditure
CIMAS: Commercial and Industrial Medical Aid Society
MAS: Medical aid societies
NAMAS: National Association of Medical Aid Societies
NHA: National Health Accounts
NHI: National Health Insurance
NHIS: National Health Insurance Scheme
OOP: Out-of-pocket
PHI: Private health insurance
PSMAS: Public Service Medical Aid Society
PSMI: Premier Service Medical Investments
THE: Total Health Expenditure
UHC: Universal Health Coverage
ZIMA: Zimbabwe Medical Association
